# Contextual influence on poor self-rated health in patients with Chagas disease: multilevel study

**DOI:** 10.1590/1413-81232022277.01682022

**Published:** 2022-02-26

**Authors:** Ariela Mota Ferreira, Ester Cerdeira Sabino, Léa Campos de Oliveira-da Silva, Cláudia Di Lorenzo Oliveira, Clareci Silva Cardoso, Antonio Luiz Pinho Ribeiro, Renata Fiúza Damasceno, Sâmara Fernandes Leite, Thallyta Maria Vieira, Maria do Carmo Pereira Nunes, Desirée Sant’ Ana Haikal

**Affiliations:** 1 Programa de Pós-Graduação em Ciências da Saúde, Universidade Estadual de Montes Claros. Av. Prof. Rui Braga s/n, Vila Mauriceia. 39401-089 Montes Claros MG Brasil.; 2 LIM46, Hospital das Clínicas, Faculdade de Medicina, Universidade de São Paulo. São Paulo SP Brasil.; 3 Grupo de Pesquisa em Epidemiologia e Novas Tecnologias em Saúde, Campus CCO, Universidade Federal de São João del-Rei. Divinópolis MG Brasil.; 4 Departamento de Medicina Interna, Universidade Federal de Minas Gerais. Belo Horizonte MG Brasil.

**Keywords:** Chagas disease, Self-rated health, Health status, Epidemiologic studies, Multilevel analysis

## Abstract

Chagas disease (CD) is recognized by the World Health Organization as one of the thirteen most neglected tropical diseases in the world. Self-perceived health is considered a better predictor of mortality than objective measures of health status, and the context in which one lives influences this predictor. This study aimed to evaluate the prevalence and individual and contextual factors associated with poor self-rated health among CD patients from an endemic region in Brazil. It is a multilevel cross-sectional study. The individual data come from a cross-section of a cohort study named SaMi-Trop. Contextual data was collected from publicly accessible institutional information systems and platforms. The dependent variable was self-perceived health. The analysis was performed using multilevel binary logistic regression. The study included 1,513 patients with CD, where 335 (22.1%) had Poor self-rated health. This study revealed the influence of the organization/offer of the Brazilian public health service and of individual characteristics on the self-perceived health of patients with CD.

## Resumo

A Doença de Chagas (DC) é reconhecida pela Organização Mundial da Saúde como uma das treze doenças tropicais mais negligenciadas do mundo. A autopercepção de saúde é considerada um melhor preditor de mortalidade do que medidas objetivas do estado de saúde, e o contexto em que se vive influencia esse preditor. O objetivo deste estudo foi avaliar a prevalência e os fatores individuais e contextuais associados à pior autopercepção em saúde de pacientes com DC de uma região endêmica do Brasil. É um estudo transversal multinível. Os dados individuais vêm de um corte transversal de um estudo de coorte denominado SaMi-Trop. Os dados contextuais foram coletados a partir de plataformas e sistemas de informações institucionais acessíveis ao público. A variável dependente foi a autopercepção de saúde. A análise foi realizada por meio de regressão logística binária multinível. Participaram do estudo 1.513 pacientes com DC, sendo 335 (22,1%) com pior autopercepção de saúde. Este estudo revelou a influência da organização/oferta do serviço público de saúde brasileiro e de características individuais na autopercepção de saúde de pacientes com DC.

## Introduction

Recognized by the World Health Organization (WHO) as one of the thirteen most neglected tropical diseases in the world^1^, Chagas disease (CD) is an infectious disease that represents a serious public health problem in Latin America. In Brazil, CD is considered one of the main medical and social problems^2^. The initial stage of infection with *Trypanosoma cruzi*, the main transmitter of CD, lasts from 4 to 8 weeks and is generally asymptomatic. About 60 to 70% of patients do not develop a clinically visible disease. However, the remaining patients (30 to 40%) develop one of the chronic forms of the disease (cardiac, digestive, or cardio-digestive) that persists during the life of the host^3^.

Self-perception of health is an important indicator used in social epidemiology. It is considered a better predictor of mortality than objective measures of health status^4,5^, since it consistently predicts functional decline^4^, in addition to influencing the frequency of seeking health care and the acceptance of treatment plans^6^. CD in its chronic form negatively impacts patients’ self-perceived health^7^, however there are few studies that investigate this topic in CD.

It is known that information related to the context in which patients live has an influence on their health conditions, as well as on their self-perception^5,8–12^. The use of multilevel models which simultaneously include context variables (social structure to which the individual belongs) in addition to conventional individual variables^13^, has been an important tool in scientific investigations, as it overcomes some limitations of traditional epidemiology when considering distinct hierarchical levels in the analyses. These models consider that attributes at the individual level may not be sufficient to explain the process of illness, since within the context there are cultural and geographical factors that can affect individuals directly or indirectly^14^.

Although the contextual influence on self-perceived health is recognized^8^, studies with this approach are scarce. In addition, no previous multilevel studies were found that investigated self-perceived health among CD patients. Thus, this study aimed to assess the prevalence and individual and contextual factors associated with poor self-rated health among patients with CD from an endemic region in Brazil.

## Methods

Ethical approval was obtained from the relevant ethic committee (CEP/USP - 042/2012, UNIMONTES 2.474.172 e CONEP 179.685). All subjects agreed to participate to this study and signed the informed consent form prior to the beginning of the study.

This is a cross-sectional study with multilevel analysis that considered individual and contextual information. The individual data came from a cross-section (follow-up) in a cohort study named SaMi-Trop (Research on Biomarkers in Neglected Tropical Diseases in São Paulo/Minas Gerais). SaMi-Trop is a multicenter study which involves the involvement of four Brazilian public universities^15^. The study was carried out in 21 municipalities selected for showing a high prevalence of CD. These municipalities belong to two macro-regions of the state of Minas Gerais endemic to CD: the northern region of Minas and the Vale do Jequitinhonha region. The contextual data used were extracted from the official database of the Brazilian government, and were collected at the municipal level.

**figure 1.**
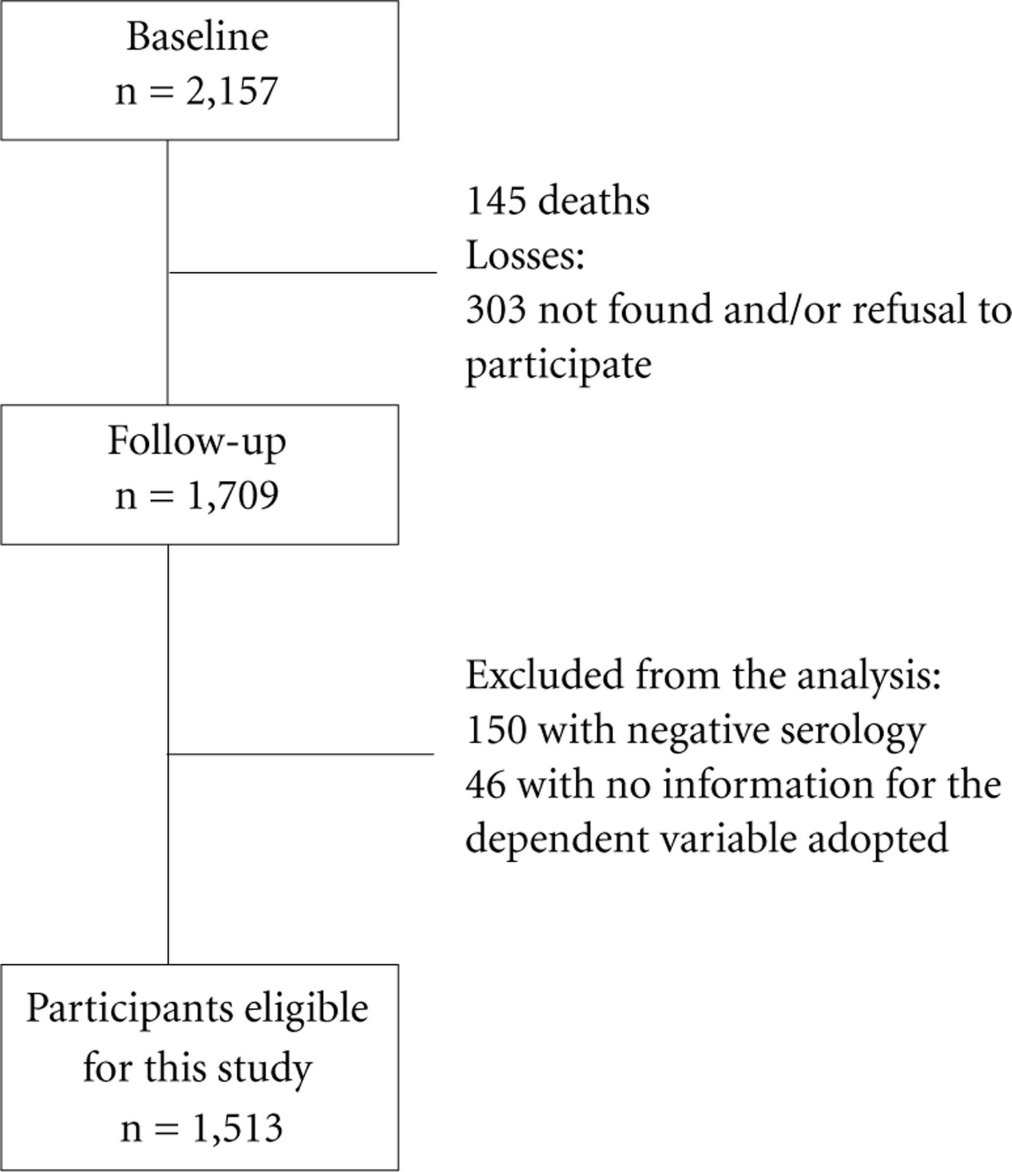
Flowchart of eligible, lost and excluded CD patients of the study. SaMi-Trop Project, Minas Gerais. Source: Authors.

The SaMi-Trop methodology has been presented in detail in previous publications^15,16^. To date, two evaluations have been carried out, the baseline and the first follow-up. The baseline was composed of 2,157 individuals. Follow-up was carried out two years later, and it was possible to collect information for 1,709 individuals, being those initially included in the cohort (79%). A total of 196 individuals were excluded (150 for not having a positive serology for the anti-*T. cruzi* antibody and 46 for not having valid information for the dependent variable adopted (Figure 1). The analyses of the present study were conducted with data from the first follow-up interview.

Baseline data collection was carried out between 2013/2014. The first follow-up visit took place between 2015/2016 where interviews were conducted with patients, with collection of peripheral blood, ECG and echocardiogram exams.

Contextual data collection was conducted for the social, economic, demographic, epidemiological, and health services characterization of the 21 municipalities included in the SaMi-Trop. Chart 1 presents these variables, the year adopted as a reference for the collection (available data that was the closest to the year of the cohort follow-up 2015/2016), its source, its concept, and the way the information was categorized to conduct the analysis.

The variables Municipal Human Development Index (MHDI) and SUS Performance Index (IDSUS) were collected, categorized according to national standard and subsequently dichotomized. The other contextual variables were collected in numerical form and later dichotomized using the 25^th^ or 75^th^ percentile as the cutoff point, depending on whether the variable represented a negative (low values indicated better situation) or positive (high values indicated better situation). The objective was to separate 25% of the better-off municipalities vs. 75% of the municipalities in the worst situation, since in general the municipalities included had similar profiles, and for the most part, were precarious (Chart 1).

The organization of variables in this study followed the conceptual theoretical model of Andersen and Davidson^17^, which considers “self-perception of health” as an outcome of interest. Following this model, the dependent variable was self-perception of health, constructed from the participant’s self-report during the interview after being asked: “How would you rate your health today?”, with a Likert scale as the answer options being adopted, and later dichotomized as “Poor” (bad and very bad) vs. “Good” (good, very good, and average). The dichotomization adopted allowed us to investigate the negative self-perception, which reflects the most critical condition of health and quality of life, and thus, fulfill the objective of the work.

**figure 2.**
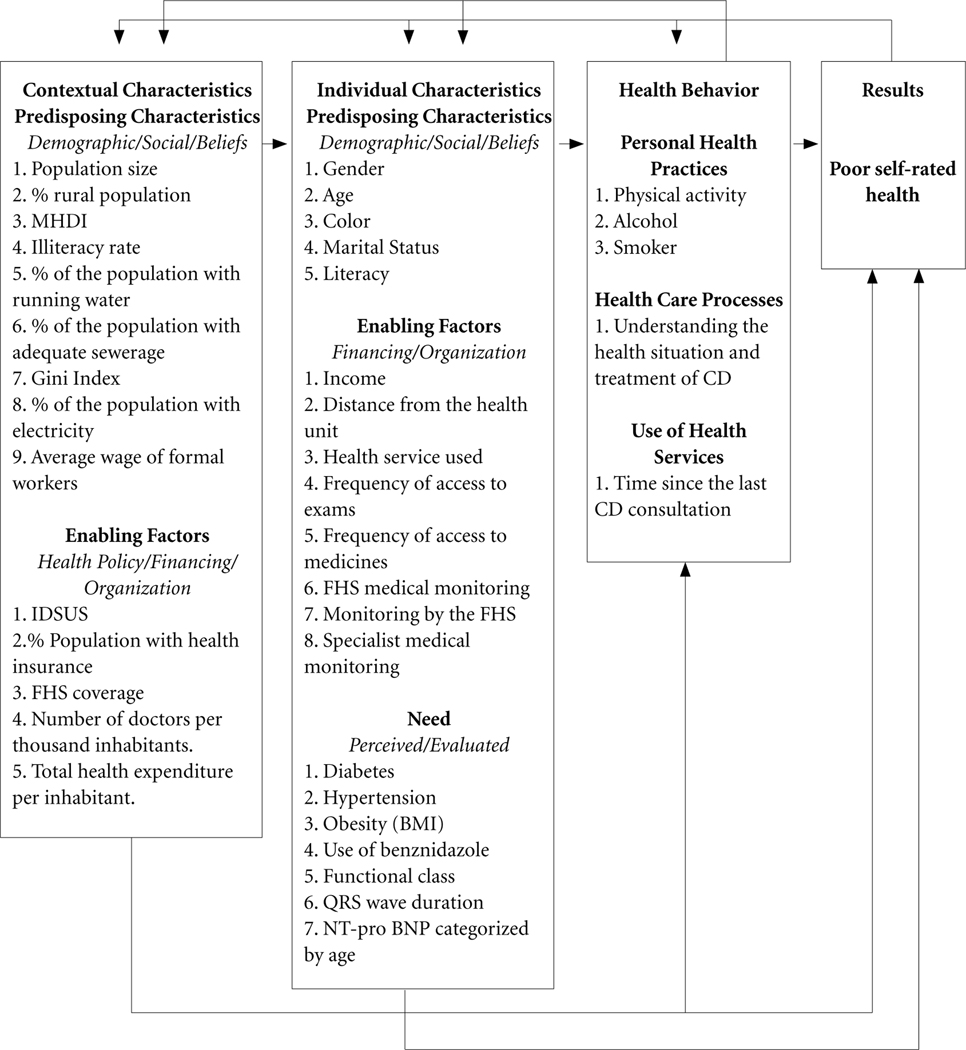
Theoretical model adopted. Source: Authors.

The independent variables were also grouped as suggested by the theoretical model adopted^17^ (Figure 2). The model has three blocks, the first block consisting of contextual variables, and the second and third blocks consisting of variables measured at the individual level: individual characteristics and health-related behavior. The information on the last two levels came from the first follow-up interview of the SaMi-Trop project.

In the 1^st^ block, contextual characteristics related to the municipalities were included considering the variables presented in Chart 1, subgrouped into 1) Predisposing Characteristics, and 2) Enabling Factors.

The 2^nd^ block (individual characteristics) considered three subgroups: 1) Predisposing Characteristics: gender (female, male), age (up to 60 years, 60 years or more), self-declared skin color (white, black, brown and others (indigenous and yellow)), marital status (stable union, without stable union), literacy (no, yes); 2) Enabling Factors: income (up to 1 minimum wage, above 1 minimum wage), dichotomized considering the value of the minimum wage in force at the time of data collection (R$ 724 - U$ 304.20), distance of residence from the Basic Heath Unit (BHU) (over 100 km, from 6 to 99 km, from 0 to 5 km), type of health service most frequently used to treat CD (none, public, private/health insurance), frequency of access to exams (rarely/ never, regularly/frequently, always), frequency of access to medications (rarely/never, regularly/frequently, always), monitoring by the FHS (not monitored, irregularly monitored, regularly monitored), specialist medical monitoring (not monitored, irregularly monitored, regularly monitored); and 3) Perceived/Evaluated Needs: self-report of diabetes diagnosis (yes, no), self-report of arterial hypertension diagnosis (yes, no), body mass index (BMI) (overweight, normal weight), previous use of Benznidazole (BZN) (no; yes), functional class (with limitations - Classes II, III and IV, without limitations - Class I)^18^. BMI was calculated from weight and height measurements using the formula: BMI=Weight (kg)/(Height)^2^ (m), individuals with normal weight were those with up to 24.9 kg/m^2^, and overweight those above that value^19^. The duration of the QRS complex (greater than or equal to 120 m/s, up to 119 m/s) and the age-adjusted NT-pro BNP^20^ (changed, not changed) were collected from ECG and blood tests, respectively. NT-pro BNP levels are quantitative plasma biomarkers of heart failure, and The QRS complex is the combination of three of the graphical deflections seen on a electrocardiogram of corresponds to the ventricle depolarization. These variables, with this cut-off point, reflect worse health conditions, with symptoms that affect the quality of daily life^20,21^.

The 3^rd^ block (health behavior) considered three subgroups: 1) Personal Health Practices: physical activity practice (no, yes); alcohol (frequent use of alcohol, infrequent use of alcohol), and smoking (smoker, non-smoker). The practice of physical activity was considered as answered (yes or no). Alcohol was measured by the question “How many times in the last thirty days, did you consume alcohol?”, the answer options were: did not consume, consumed less than once a week, consumed 1 to 2 times per week, consumed 3 to 5 times a week, and consumed every day. The answers to this question were dichotomized and grouped into two categories: infrequent use (did not consume/consumed less than once a week/consumed 1 to 2 times a week) vs. frequent use (consumed 3 to 5 times a week/consumed every day). Smoking was assessed by the question: “Which of the following phrases best defines your habits in relation to cigarette use?”, with the answer options being: I have never smoked, I have smoked, but I don’t smoke anymore, or I currently smoke. Smokers were considered to be those who smoked at the time of data collection and ex-smokers and those who had never smoked were grouped in the non-smoking category; 2) Health Care Processes: understanding the health situation and treatment of CD, as assessed by the question “Do you consider that you understand your health situation and the care you should take during your treatment for Chagas disease?” (I don’t understand enough, I understand reasonably, I understand well); and 3) Use of Health Services: time since the last consultation for CD, measured by the question “How long has it been since your last medical consultation related to Chagas disease?”, the answer being numerical and subsequently dichotomized into more than one year vs. a year or less.

Initially, a descriptive analysis of all variables was conducted. Simple (n) and relative (%) frequencies were estimated for each category of variables. For the age variable, the mean and its standard deviation were also estimated. In addition, the description of contextual variables according to self-rated health was presented.

Subsequently, bivariate analyzes were conducted between the investigated outcome and the individual variables. For this, Pearson’s Chisquare test was used. In the multiple analysis, multilevel binary logistic regression was adopted, so that the variables were introduced into the model by levels of grouping (3 levels), according to the theoretical model adopted. Initially, all contextual variables (1^st^ level) were introduced and the model was adjusted to a significance level of 5%, following the backward manual modeling technique. Subsequently, maintaining the variables of the first level, the individual variables (2^nd^ level) were introduced from the screening obtained by the bivariate analysis (variables with p value≤0.20). The model was adjusted again. Finally, there was the introduction of individual health behavior variables (3^rd^ level) also screened by bivariate analysis and a new model adjustment was performed. The multilevel analysis used the fixed effects model (intercept model) to estimate the fit between the outcome and the contextual and individual explanatory variables with the mixed coefficients and logit function to obtain the odds ratios (OR) and confidence interval measures (CI) 95%. The model was adjusted to each level introduced in a hierarchical manner, with only variables with statistical significance remaining. The deviance statistic represented by “−2 loglikelihood”, was the indicator used to assess the fit quality measure, making it possible to compare the likelihood functions. The analyzes were performed using Predictive Analytics SoftWare (PASW/SPSS)® version 18.0 for Windows® and STATA, version 17 (StatCorp, College Station, Texas, USA)®, statistical software.

## Results

The Descriptive and bivariate analysis of contextual characteristics and their association with self-rated health in patients with Chagas disease is shown in Table 1.

Of the 1,513 CD patients participating in this study, 335 (22.1% 95%CI=20.0–24.2) showed poor self-rated health. The average age of the participants was 59.9 (±12.2) years, the majority were female (67.9%), brown (59.1%), and with a monthly income up to one minimum wage (53%). Among the municipalities studied, the poor self-rated health ranged from 6.7% to 57.1%. The distribution of CD patients according to individual characteristics and health behaviors is shown in Table 2.

In the bivariate analysis, the individual variables screened to compose the initial multiple model (p≤0.20) were: gender, age, literacy, family income, distance from the BHU, health service used, frequency of tests, frequency of access to medication, medical monitoring by the FHS, specialist monitoring, diabetes mellitus, hypertension, use of BZN in the last 2 years, functional class, NT–pro BNP, physical activity, smoking, understanding of CD, and time since the last CD visit (Table 2).

The final adjusted multiple model revealed that among the contextual characteristics, there was less odds of poor self-rated health among those who lived in municipalities with a smaller population when compared to those who lived in municipalities with a larger population (OR=0.6; 95%CI=0.3–0.9), and a greater odds among those who lived in municipalities with a higher illiteracy rate when compared to those who lived in municipalities with a lower illiteracy rate (OR=1.5; 95%CI=1.0–2.4) and. among those who lived in municipalities with fewer doctors per thousand inhabitants when compared to those who lived in municipalities with a higher doctors per thousand inhabitants (OR=1.5; 95%CI=1.0–2.4). Among the variables of the second level, there were greater odds of poor self-rated health among those with limitations in functional class when compared to those without limitations in functional class (OR=2.0; 95%CI=1.4–2.7), with a changed level of NT-pro BNP adjusted for age when compared to those not changed (OR=1.9; 95%CI=1.2–2.9), who reported arterial hypertension when compared to those without arterial hypertension (OR=1.5; 95%CI=1.0–2.1), who had an income below one minimum wage when compared to those income above one minimum wage (OR=1.5; 95%CI=1.1–2.0), who lived more than 100 km from the BHU when compared to those who lived 0 to 5 km from the BHU (OR=2.5; 95%CI=1.3–4.5), and among those who reported having irregular FHS monitoring when compared to those without reported having regularly FHS monitoring (OR=1.7; 95%CI=1.1–2.6). Among the variables of the third level, greater odds of poor self-rated health were observed among those who did not practice physical activity when compared to those that practice physical activity (OR=1.8; 95%CI=1.2–2.7) and who smoked when compared to those that did not smoke (OR=2.6; 95%CI=1.4–4.7) (Table 3).

## Discussion

This study showed a prevalence of poor self-rated health of more than 22% among the individuals with CD investigated, being associated with contextual variables such as population size, illiteracy rate, and number of doctors per thousand inhabitants; and with the individual variables income, distance from the BHU, FHS monitoring, arterial hypertension, functional class, NT-pro BNP level, physical activity and smoking.

The high prevalence of poor self-rated health among individuals with CD may be associated with the greater severity of CD in the chronic cardiac form^22^. Studies point to a wide variation in the prevalence of poor self-rated health among different populations^7,23–25^. Among patients with arterial hypertension, a prevalence of 10.4%^23^ was found, among the elderly, 13.5%^24^, and among patients with CD a prevalence of 32.8%^7^. The dependent variable, determined by means of a simple question, represents an indicator considered robust and consistent for predicting mortality and functional decline^4,5^.

Despite the recognized relevance of this indicator in chronic diseases^4,26^, there is a gap in the literature regarding the assessment of self-perceived health in patients with chronic CD, especially considering characteristics of the context where they live. To date, no previous studies have been identified that have performed a multilevel assessment related to self-perceived health among patients with CD, making comparisons of this nature impossible. The only study identified regarding self-perceived health among CD patients was conducted considering only the individual level^7^, not considering the context where the individuals lived. It is already known that the context determines the occurrence and worsening of CD, considering that most patients live in a situation of social vulnerability, with unfavorable sociodemographic, economic, and life conditions. In addition, many patients live in remote regions and have difficulty accessing specialized health services^16,27–29^.

In our study, individuals with CD who lived in cities with a smaller population were less likely to report poor self-rated health. In Brazil, the expansion of primary health care (PHC) through the FHS has increased and facilitated access to scheduling appointments and exams, especially in smaller municipalities^30^, where residents and health workers know each other and maintain greater proximity. It is believed that in smaller municipalities, the humanization of assistance is facilitated due to the closer relationship between health workers and the reality experienced by the user, which favors the construction of friendly and trusting relationships based on welcoming, bonding, listening, and dialogue^31^. It is known that health services with such characteristics bring greater satisfaction to their users^32^. Satisfaction with health services is associated with greater positive self-perception of health^33^. A previous study found that health services in rural areas were better evaluated than those in urban areas^34^. Possibly in smaller municipalities and in rural and remote areas, there is greater resignation to the health conditions experienced, increasing positive self-perception^35^.

The higher illiteracy rate was another contextual variable that remained in the final model associated with poor self-rated health among CD patients. It is already agreed that the level of education is one of the definers of the conduct that the individual takes within the health-disease process^36^. Health is influenced by educational level, with lower education associated with greater population illness^37^. Previous studies, including a systematic review, have pointed out the influence of schooling on the self-perception of health of other populations^38,39^. However, no studies have been identified that evaluated this relationship between patients with CD.

In this study, individuals with CD who lived in municipalities with fewer doctors per thousand inhabitants had greater odds of poor self-rated health. The WHO does not recommend or establish adequate rates of doctors per number of inhabitants, as this parameter depends on regional, socioeconomic, cultural, and epidemiological factors. Thus, there would be little point in establishing a generalized “ideal rate” for all countries^40^. Despite this, this indicator has been used due to the lack of any other that considers the complexity of care models^41^. Brazil still has one of the lowest rates of doctors per inhabitants in the world, and in January 2018 the country had 2.18 doctors per thousand inhabitants, while the average number for countries included in the Organization for Economic Cooperation and Development (OECD) is of 3.4 doctors per thousand inhabitants, reaching up to 5.1 doctors per thousand inhabitants in countries such as Norway^42^. The municipalities where the participants of this study lived had an average of 0.68 (±0.383) doctors per thousand inhabitants, and when categorized according to the percentile proposed in the study, the municipalities in the category “lesser number of doctors” had less than 0.79 doctors per thousand inhabitants, thus being well below the national average. The existence of a referral doctor for a given community indicates the possibility of establishing a bond, and consequently, strengthening PHC attributes such as longitudinality and coordination of care^43^. Access and continuity of the PHC service is associated with better self-perceived health^25^. This association shows that the simple quantitative – the presence and permanence of doctors in the municipality – influence the self-perception of health among patients with CD.

The individual variables that reflect living conditions associated with poor self-rated health of patients with CD were income, distance from home to the BHU, and monitoring by the FHS. The distribution of income within a society is a health predictor^44^, and this relationship between the lowest income and the worst self-perception of health is already known^10,25,38^.

The greater distance between the home of patients with CD and the BHU suggests issues related to access to PHC services. Previous studies have already found that access to health services is influenced by distance^45,46^. Users who most frequent the BHU are those who live in its vicinity, which facilitates the link between patients and service^46^, which can influence self-perceived health.

Likewise, FHS monitoring was also associated with the outcome. This variable also reflects access to health services. There were greater odds of poor self-rated health among patients with CD who report irregular monitoring by the FHS. The FHS represents the “gateway” of SUS, the current public health model in force in Brazil, for access to PHC^47^. The difficulty in accessing health services is associated with poor self-rated health. Previous studies have identified such an association among the elderly^24^ and in the general population^25^.

Although the influence of variables that reflect health conditions on self-perception is already established in the literature^24,48–51^, our study confirmed this finding, but innovated when considering markers of CD severity. No previous studies have been identified that have assessed the relationship between such markers and self-perceived health. Regarding the presence of systemic arterial hypertension, other studies have already shown its association with poor self-rated health^24,50^. The limited functional class and the altered NT-pro BNP level negatively influenced self-perceived health among CD patients. This finding corroborates the robustness of the dependent variable as a health predictor. The most advanced functional class is associated with worse health conditions^29,48^, as it reflects the extent of symptoms of heart failure, common in CD. The levels of NT-pro BNP are also accurate discriminators of the diagnosis of heart failure, powerful predictors of death, and assist in the risk stratification of patients^29,49^, a frequent situation due to CD. It has been verified that the functional class with limitations and the altered NT-pro BNP level were associated with a worse cardiac prognosis in CD, increasing the odds of pacemaker implantation, atrial fibrillation and/or death in two years of monitoring^29^.

The health behavior variables that were associated with poor self-rated health of CD patients were physical inactivity and smoking, a category most strongly associated with the outcome. The adoption of healthier lifestyles suggests greater self-care in health, and consequently, better self-perception of one’s own health. Other studies have also found that poor self-rated health is strongly associated with physical inactivity^52,53^, as well as smoking^54,55^.

Regarding the limitations, in addition to the cross-sectional design that does not allow establishing causal relationships, there is a limitation regarding the extrapolation of the results to other populations with CD, who live in different contexts to those portrayed in this investigation. However, it has already been observed that populations with CD generally have a similar epidemiological profile^48^. On the other hand, the large sample size of patients with CD and who live in endemic areas of small municipalities is a strong point of our study, as it portrays scenarios commonly overlooked in the investigations. In addition, the results were reliably measured, reflecting the patients’ social and clinical conditions, as well as their parasitological status. Even though some of the information collected came from self-reporting, which can lead to measurement bias, high accuracy of self-reported questions for chronic conditions has already been verified^56^. Self-perceived health proved to be an important indicator to be used in health planning and clinical evaluation. This indicator was sensitive to the contextual and individual conditions of patients with CD and deserves to be considered in global assessments of these individuals.

This self-perception was influenced by the context where individuals lived, even after adjustment for important individual markers. The odds of poor self-rated health were lower among residents of municipalities with a smaller population size. On the other hand, the odds of poor self-rated health were greater among residents of municipalities with higher illiteracy rates and with a lower ratio of doctors per inhabitant. At the individual level, the poor self-rated health among patients with CD was influenced by sociodemographic issues, access to health services, clinical/laboratory issues, and behaviors. Thus, we observe the influence of the organization/offer of the Brazilian public health service and of individual characteristics in the self-perception of health of patients with CD.

Our findings also corroborate the robustness of the dependent variable as a predictor of health conditions, since important clinical and laboratory markers related to the severity of CD remained in the final model. Despite its simplicity of measurement, self-perception of health proved to be a sensitive indicator of health status in CD, deserving greater recognition both among scientific studies and in the conduct of clinical practices, which may favor not only the implementation of care, but also its management.

## Figures and Tables

**Chart 1.** Contextual variables collected from publicly accessible institutional information systems and platforms, according to the year, source, concept, and cut-off point adopted for categorizing the variable.

Contextual variables	Year of collection	source	Concept	Cut off point adopted
1. Total population	2010	Atlas of Human Development in Brazil^1^	Number of people residing in the municipality.	75^th^ percentile=31,003 inhabitants.
2. % of the rural population	2010	Atlas of Human Development in Brazil^1^	Percentage of people residing in the area outside urban limits.	25^th^ percentile=33.11%
3. Municipal Human Development Index (MHDI)	2010	Atlas of Human Development in Brazil^1^	Geometric average of the indices of the dimensions Income, Education and Longevity, with equal weights. This varies from 0 to 1, with higher values indicating a better situation.	Categorized by international standard and dichotomized as low (<0,550) vs. high (>0,700) /medium (0,551–0,699)
4. Gini index	2010	Atlas of Human Development in Brazil^1^	Measures the degree of inequality in the distribution of household income per capita. Its value ranges from 0, when there is no inequality, to 1 when the inequality is maximum.	25^th^ percentile=0.4642
5. Average wage of formal workers	2015	IBGE^2^	Average wage of formal workers, measured in number of minimum wages.	75^th^ percentile=1.7 minimum wages
6. Illiteracy rate	2010	DATASUS^3^	Percentage of illiterates in the population of the municipality	25^th^ percentile=17.1%
7. % of the population with running water	2010	Atlas of Human Development in Brazil^1^	Percentage of the population of the municipality with access to running water	25^th^ percentile=85.4%
8. % of the population with adequate sewerage	2010	Atlas of Human Development in Brazil^1^	Percentage of the population of the municipality with access to adequate sewerage	25^th^ percentile=49.5%
9. % of the population with electricity	2010	Atlas of Human Development in Brazil^1^	Percentage of the population of the municipality with access to electricity	25^th^ percentile=98.8%
10. SUS Performance Index (IDSUS)	2010	Performance Index of the Public Health System^4^	Assesses the municipal performance of the SUS* regarding: universality of access, integrality, equality, resolvability and equity of care, decentralization with a single command by management level, tripartite responsibility, regionalization and hierarchy of the health service network.	Categorized by national standard and dichotomized as 0.500–0.599 (low) vs. 0.600–0.699/0.700–0.799 (high/medium)
11. Total health expenditure per inhabitant	2016	SIOPS - Public Health Budget Information System^5^	Measures the dimension of total public health expenditure per inhabitant	75^th^ percentile=R$ 610.72
12. Number of doctors per thousand inhabitants	2017	CNES - National Health Establishment Register^6^	Number of doctors per thousand inhabitants present in the municipality hired by the SUS	75^th^ percentile=0.79
13. % of the population with health insurance	2017	Department of Primary Care - Ministry of Health^7^	Proportion of population of the municipality with health insurance	75^th^ percentile=3.03%
14. FHS coverage	2017	Department of Primary Care - Ministry of Health^7^	Percentage of coverage of the population of the municipality by teams of the family health strategy (FHS).	75^th^ percentile=100%

* SUS=Sistema Único de Saúde (Brazilian Public Health System).

1 http://www.atlasbrasil.org.br/2013/pt/o_atlas/idhm/.

2 http://www.censo2010.ibge.gov.br/sinopse/index.php?uf=31&dados=0.

3 http://www2.datasus.gov.br/DATASUS/index.php?area=02.

4 http://idsus.saude.gov.br/mapas.html.

5 http://siops-asp.datasus.gov.br/CGI/deftohtm.exe?SIOPS/serhist/municipio/indicMG.def.

6 http://cnes.datasus.gov.br/.

7 http://sisaps.saude.gov.br/notatecnica/frmListaMunic.php.

Source: Authors.

**Table 1.** Descriptive analysis of contextual characteristics and their relation with self-rated health in patients with Chagas disease (CD) (n=1,513).

Contextual variables	Descriptive		Bivariate	

	n municipalities	n individuals	self-rated health	

			Poor n (%)	95%CI
Total population				
<31,003 inhabitants	16	902	196 (21.7%)	19.6–23.77
>31,003 inhabitants	5	611	139 (22.7%)	20.58–24.81
% of the rural population				
<33.11%	5	529	104 (19.7%)	17.69–21.70
>33.12%	16	984	231(23.5%)	21.36–25.63
Municipal Human Development Index. (MHDI)				
Low	4	1,256	61 (23.7%)	21.55–25.84
High/medium	17	257	274 (21.8%)	19.71–23.88
Gini index				
<0.4642	5	229	286 (22.3%)	20.20–24.39
>0.4643	16	1,284	49 (21.4%)	19.71–23.88
Average wage of formal workers				
<1.7 minimum wages	12	780	172 (22.1%)	20.00–14.19
>1.8 minimum wages	9	733	163 (22.2%)	20.10–24.19
Illiteracy rate				
<17.1%	5	667	139 (20.8%)	18.75–22.84
>17.2%	16	846	196 (23.2%)	21.07–25.32
% of the population with running water				
<85.4%	16	1,136	254 (22.4%)	20.29–24.50
>85.5%	5	377	81 (21.5%)	19.42–23.57
% of the population with adequate sewerage				
<49.5%	16	1,100	263 (23.9%)	21.75–26.04
>49.6%	5	413	72 (17.4%)	15.48–19.31
% of the population with electricity				
<98.8%	16	1,209	274 (22.7%)	20.58–24.81
>98.8%	5	304	61 (20.1%)	18.08–22.11
SUS Performance Index (IDSUS)				
0.500–0.599	7	604	152 (25.2%)	23.01–27.38
0.600–0.799	14	909	183 (20.1%)	18.08–22.11
Total health expenditure per inhabitant				
<R$ 610.72	16	1,142	269 (23.6%)	21.65–25.94
>R$ 610.73	5	371	66 (17.8%)	15.87–19.72
Number of doctors per thousand inhabitants				
<0.79	16	1,100	262 (23.8%)	21.65–25.94
>0.80	5	413	73 (17.7%)	15.87–19.72
% of the population with health insurance				
<3.03%	16	978	225 (23%)	20.87–25.12
>3.04%	5	535	110 (20.6%)	18.56–22.63
FHS coverage				
<99%	4	587	202 (21.8%)	19.71–23.88
100%	17	926	133 (22.7%)	20.58–24.81

Source: Authors.

**TABLE 2.** Descriptive and bivariate analysis of individual characteristics and health behavior and their association with self-rated health in patients with Chagas disease (CD) (n=1,513).

Characteristics	Descriptive	Bivariate		P-value

		Self-rated health		

	n	Poor n (%)	95%CI	
Individual				
Gender				
Female	1,028	241 (23.4%)	21.2–25.5	0.076
Male	485	94 (19.4%)	17.4–21.39	
Age				
60 years or older	667	137 (20.5%)	18.46–22.53	0.183
Up to 60 years	846	198 (23.4%)	21.26–25.53	
Self-reported skin color*				
White	321	236 (73.5%)	71.27–75.72	0.424
Brown	891	650 (73%)	70.76–75.23	
Black	270	209 (77.4%)	75.29–79.50	
Others	26	21 (80.8%)	78.81–82.78	
Marital status*				
Single. widowed or divorced	526	116 (22.1%)	20.00–24.19	0.956
Married or cohabiting	983	218 (22.2%)	20.10–24.29	
Literacy*				
No	645	157 (24.3%)	22.13–26.46	0.076
Yes	863	177 (20.5%)	18.46–22.53	
Family income*				
Up to R$ 727.00	800	200 (25%)	22.81–27.18	0.004
Above R$ 728.00	709	134 (18.9%)	16.92–20.87	
Distance from the Health Unit*				
Over 100 km	61	27 (44.3%)	41.79–46.80	<0.001
6 to 99 km	322	71 (22%)	19.91–24.08	
0 to 5 km	736	151 (20.5%)	18.46–22.53	
Health service used*				
None	132	39 (29.5%)	27.02–31.79	0.090
Public	1,013	214 (21.1%)	19.04–23.15	
Private/health insurance	368	82 (22.3%)	20.20–24.39	
Examination frequency*				
Rarely or never	758	190 (25.1%)	22.91–27.28	0.004
Regularly/frequently	648	132 (20.4%)	18.36–22.43	
Always	107	13 (12.1%)	10.45–13.74	
Frequency of access to medicines*				
Rarely or never	665	153 (23%)	20.87–25.12	0.056
Regularly/frequently	421	97 (23%)	20.87–25.12	
Always	317	53 (16.7%)	14.82–18.57	
Medical monitoring by the FHS*				
Not monitored	675	138 (20.4%)	18.36–22.43	<0.001
Monitored irregularly	435	123 (28.3%)	26.03–30.56	
Monitored regularly	349	56 (16%)	14.15–17.84	
Monitoring by specialist *				
Not monitored	905	184 (20.3%)	18.27–22.32	0.092
Monitored irregularly	304	80 (26.3%)	24.08–28.51	
Monitored regularly	237	52 (21.9%)	19.81–23.98	
Diabetes mellitus				
Yes	176	47 (26.7%)	24.47–28.92	0.121
No	1,337	288 (21.5%)	19.42–23.57	
Arterial hypertension				
Yes	982	233 (23.7%)	21.55–25.84	0.043
No	531	102 (19.2%)	17.21–21.18	
BMI*				
Overweight	796	174 (21.9%)	19.81–23.98	0.802
Normal weight	692	155 (22.4%)	20.29–24.50	
Benznidazole use in the last 2 years*				
No	1,403	303 (21.6%)	19.52–23.67	0.152
Yes	93	26 (28%)	25.73–30.26	
Functional class*				
With limitations	629	179 (28.5%)	26.22–30.77	<0.001
No limitations	870	151(17.4%)	15.48–19.31	
QRS wave duration*				
Greater than or equal to 120 m/s	590	137 (23.2%)	21.07–25.32	0.516
Up to 119 m/s	886	193 (21.8%)	19.71–23.88	
NT-pro BNP level*				
Changed	178	61 (34.3%)	31.90–36.69	<0.001
Not changed	1,277	255 (20%)	17.98–22.01	
Health behavior				
Physical activity				
No	1,153	287 (24.9%)	22.72–27.07	<0.001
Yes	360	48 (13.3%)	11.58–15.01	
Alcohol*				
Frequent alcohol use	29	5 (17.2%)	15.29–19.10	0.524
Infrequent alcohol use	1,482	329 (22.2%)	20.10–24.29	
Smoking*				
Smoker	89	33 (37.1%)	34.66–39.53	<0.001
Never smoked or ex-smoker	1,423	301 (21.2%)	19.14–23.25	
Understanding CD*				
Do not understand enough	707	200 (28.3%)	26.03–30.56	<0.001
Reasonable understanding	473	67 (14.2)	12.44–15.95	
Understands well	193	21 (10.9%)	9.32–12.47	
Time since the last CD consultation*				
More than a year	272	43 (15.8%)	13.96–17.63	0.003
One year or less	881	215 (24.4%)	22.23–26.56	

* Variation of the number of 1,513 due to loss of information.

Source: Authors.

**TABLE 3.** Final model of the Hierarchical Multilevel Logistic Regression Analysis of the factors associated with the self-rated health of the patient with Chagas disease. Minas Gerais, Brazil.

models	variables	Gross OR (95%CI)	Gross P-value	Adjusted OR (95%Ci)	P-value
Model 1 Contextual Characteristics	Population				
	Larger population	1		1	
	Smaller population	0.333 (0.141–0.786)	0.012	0.600 (0.379–0.949)	0.029
	Illiteracy rate				
	Lower illiteracy rate	1		1	
	Higher illiteracy rate	4.871 (1.506–15.751)	0.008	1.558 (1.004–2.417)	0.048
	Number of doctors per thousand inhabitants				
	Higher number of doctors	1		1	
	Lower number of doctors	1.388 (0.878–2.194)	0.160	1.512 (1.004–2.417)	0.019

Deviance (−2log Log likelihood )=1.586					

Model 2 Contextual Characteristics Individual Characteristics	Income				
	Greater than one wage	1		1	
	Less than/lower one wage	1.401 (0.984–1.995)	0.055	1.523 (1.158–2.003)	0.003
	Distance from the BHU				
	0 to 5 km	1		1	
	6 to 99 km	1.0032 (0.705–1.509)	0.733	1.050 (0.736–1.496)	0.787
	Above 100 km	2.482 (1.286–4.791)	0.005	2.529 (1.394–4.590)	0.002
	FHS monitoring				
	Regularly	1		1	
	Irregularly	1.612 (1.012–2.568)	0.038	1.752 (1.148–2.674)	0.009
	Not monitored	1.367 (0.835–2.238)	0.210	1.368 (0.901–2.079)	0.141
	Arterial hypertension				
	Absent	1		1	
	Present	1.504 (1.022–2.215)	0.031	1.500 (1.057–2.131)	0.023
	Functional class				
	Without limitations	1		1	
	With limitations	1.861(1.331–2.601)	<0.001	2.000 (1.468–2.725)	<0.001
	NT-pro BNP level				
	Not changed	1		1	
	Changed	1.985 (1.244–3.165)	0.003	1.911 (1.256–2.906)	0.002

Deviance (−2log Log likelihood )=1.017					

Model 3 Contextual Characteristics Individual Characteristics Health Behaviors	Physical activity practice				
	Yes	1		1	
	No	1.863 (1.205–2.882)	0.002	1.853 (1.231–2.789)	0.003
	Smoking				
	No	1		1	
	Yes	3.303 (1.564–5.766)	0.002	2.621 (1.461–4.702)	0.001

Deviance (−2log Log likelihood )=996.725				

Source: Authors.
